# Humoral and cellular responses after COVID-19 booster vaccination in patients recently treated with anti-CD20 antibodies

**DOI:** 10.1038/s41408-023-00792-z

**Published:** 2023-01-23

**Authors:** Masashi Nishikubo, Yoshimitsu Shimomura, Ryusuke Yamamoto, Satoshi Yoshioka, Hayato Maruoka, Seiko Nasu, Tomomi Nishioka, Kenji Sakizono, Satoshi Mitsuyuki, Tomoyo Kubo, Naoki Okada, Daishi Nakagawa, Kimimori Kamijo, Hiroharu Imoto, Yuya Nagai, Nobuhiro Hiramoto, Noboru Yonetani, Tadakazu Kondo, Chisato Miyakoshi, Asako Doi, Takayuki Ishikawa

**Affiliations:** 1 Department of Hematology, Kobe City Medical Center General Hospital, Kobe, Hyogo 650-0047 Japan; 2 Department of Environmental Medicine and Population Science, Graduate School of Medicine, Osaka University, Osaka, 565-0871 Japan; 3 Department of Clinical Laboratory, Kobe City Medical Center General Hospital, Kobe, Hyogo 650-0047 Japan; 4 LSI Medience Laboratory, Kobe City Medical Center General Hospital, Kobe, Hyogo 650-0047 Japan; 5 Department of Research Support, Center for Clinical Research and Innovation, Kobe City Medical Center General Hospital, Kobe, Hyogo 650-0047 Japan; 6 Department of Infectious diseases, Kobe City Medical Center General Hospital, Kobe, Hyogo 650-0047 Japan

**Keywords:** Infectious diseases, B-cell lymphoma

Patients with hematologic diseases receiving anti-CD20 monoclonal antibodies show a low seroconversion rate after two doses of mRNA Coronavirus disease-2019 (COVID-19) vaccines, especially within 12 months of their last anti-CD20 antibodies [1, 2]. In early 2022, the Centers for Disease Control and Prevention recommended a third dose (booster) at least 3 months after the first two doses for immunocompromised patients [3]. Previous reports have shown that some non-responders achieved a humoral response after a third dose, but the seroconversion rate remained low at ~20% [4–7]. In these studies, the interval between a third vaccination and the last anti-CD20 antibody administration was shorter than 6 months, suggesting that the unsatisfactory seroconversion rate may be due to a lack of B cell recovery [4–7]. Conversely, a third vaccination at longer intervals may induce a higher humoral response rate in patients recently treated with anti-CD20 antibodies [6, 8]. In Japan, a third dose (booster) was offered in early 2022, 7 months after the second vaccination, regardless of the immunosuppressive status. This may allow patients to achieve B cell recovery, resulting in better humoral responses. Conversely, T-cell responses against the severe acute respiratory syndrome coronavirus 2 (SARS-CoV-2) has been identified in seronegative patients after two doses of the vaccine. Although cellular immune responses are essential for controlling viral replication after infection and preventing progression to severe diseases [9], studies focusing on T-cell responses after a third vaccination in the vulnerable population remain limited [4, 7]. Therefore, this study aims to investigate humoral and cellular responses among patients who recently received anti-CD20 antibodies to mRNA COVID-19 vaccines administered at longer intervals than those in Western countries.

This prospective study investigated humoral and cellular responses and safety after a third dose (booster) of the mRNA COVID-19 vaccines in patients with hematologic diseases at Kobe City Medical Center General Hospital. Patients were recruited between January and July 2022. In this trial, adult patients with non-Hodgkin B-cell lymphoma, idiopathic thrombocytopenic purpura (ITP), or acquired thrombotic thrombocytopenic purpura (TTP) were eligible if they received their last dose of rituximab or obinutuzumab within 12 months before two doses of SARS-CoV-2 vaccine. We excluded patients with a known and undocumented history of SARS-CoV-2 infection and comorbidities of autoimmune diseases, except ITP and TTP. We also included adult healthy volunteers without a history of COVID-19 to serve as the control group. The institutional review board approved this study and informed consent was obtained from all patients and volunteers. The blood serum of the study participants was collected before and 14–90 days after the third vaccination. We extracted relevant data from the medical records. All participants answered a questionnaire asking about local or systemic adverse events within seven days after the third vaccination. The primary endpoint was set as the proportion of subjects who acquired anti-S1 IgG antibodies. We also evaluated anti-S1 IgG titers, T-cell responses against SARS-CoV-2, and adverse events after vaccination as secondary endpoints. The details of the data, the kit used for antibody detection and T-cell responses, and statistical issues are described in the supplemental data.

Sixty-three patients with hematologic diseases who received anti-CD20 antibodies and 30 healthy controls were enrolled in the study. Fifty-three patients and all the healthy controls were included in the final analysis. Ten patients were excluded from the final analysis: four patients were infected with SARS-CoV-2 before the completion of blood sampling, two patients had undocumented COVID-19 infection, two did not have blood sampling before the third vaccination, and two did not receive their third dose. Blood samples have collected a median of ten (interquartile, IQR 4–27) days before the third vaccination and a median of 228 (IQR 202–245) days after the second vaccination. The characteristics of patients and healthy volunteers are presented in Table 1. Twenty-two (42%) patients were diagnosed with diffuse large B-cell lymphoma, 17 (32%) with follicular lymphoma, 12 (22%) with other B-cell neoplasms, and two with benign hematologic diseases. Fifty (93%) patients received BNT162b2, and 4 (7%) received mRNA-1273 as the primary two doses of vaccination. Forty (74%) patients received BNT162b2, and 14 (26%) received mRNA-1273 as their third dose. All participants received the original monovalent COVID-19 vaccines for their third doses. The median times between the last anti-CD20 antibody administration and the first and third vaccinations were 68 (IQR 23–140) and 311 (IQR 250–386) days, respectively. The median time between the second and the third vaccination was 230 (IQR 214–241) days.

**Table 1:** Characteristics of patients and healthy volunteers.

	Patient (*n* = 53)	Healthy volunteer (*n* = 30)
Age, median (IQR), years	73 (68, 77)	32 (26, 41)
Sex, *n* (%)		
Male	31 (58%)	9 (30%)
Female	22 (42%)	21 (70%)
Vaccine, *n* (%)		
PP-P	38 (72%)	30 (100%)
PP-M	11 (21%)	0 (0%)
MM-P	2 (3.8%)	0 (0%)
MM-M	2 (3.8%)	0 (0%)
Diseases, *n* (%)		
DLBCL, NOS	22 (42%)	NA
FL	17 (32%)	
Other B-cell malignancies^a^	12 (22%)	
Benign hematologic diseases^b^	2 (3.8%)	
Disease status, *n* (%)		
Complete remission	44 (83%)	NA
Not in complete remission	2 (3.8%)	
On active therapy^c^	7 (13%)	
Previous use of bendamustine*, n (%)*	23 (43%)	NA
IgG, median (IQR), mg/dL	883 (696, 1084)	1 108 (1029, 1386)
>700 mg/dL, *n* (%)	39 (74%)	30 (100%)
≤700 mg/dL, *n* (%)	14 (26%)	0 (0%)
IgA, median (IQR), mg/dL	135 (79, 236)	184 (150, 239)
>80 mg/dL, *n* (%)	38 (72%)	30 (100%)
≤80 mg/dL, *n* (%)	15 (28%)	0 (0%)
IgM, median (IQR), mg/dL	33 (20, 65)	15 (73, 141)
>40 mg/dL, *n* (%)	22 (42%)	29 (97%)
≤40 mg/dL, *n* (%)	31 (58%)	1 (3.3%)
WBC, median (IQR) × 10^3^/μL	5.000 (4.200, 6.400)	6.750 (5.125, 7.850)
Lymphocytes, median (IQR) × 10^3^/μL	1.396 (0.936, 1.755)	1.596 (1.333, 1.977)
>1.0 × 10^3^/μL, *n* (%)	36 (67%)	30 (100%)
≤1.0 × 10^3^/μL, *n* (%)	18 (33%)	0 (0%)
B cells, median (IQR)/μL	0 (0, 168)	247 (184, 314)
B-cell fraction, *n* (%)		
>3%	23 (43%)	30 (100%)
≤3%	31 (57%)	0 (0%)
T cells, median (IQR)/μL	896 (635, 1305)	1 139 (881, 1397)
CD4+ T cells, median (IQR)/μL	334 (224, 507)	638 (518, 835)
>0.4 × 10^3^/μL, *n* (%)	21 (40%)	29 (97%)
≤0.4 × 10^3^/μL, *n* (%)	32 (60%)	1 (3.3%)
CD8+ T cells, median (IQR)/μL	483 (310, 764)	421 (325, 522)
>0.4 × 10^3^/μL, *n* (%)	33 (62%)	16 (53%)
≤0.4 × 10^3^/μL, *n* (%)	20 (38%)	14 (47%)
NK cells, median (IQR)/μL	185 (122, 400)	221 (166, 293)
Time from last anti-CD20 antibody treatment to third vaccination, median (IQR), days	320 (250, 389)	NA
Over 9 months	34 (64%)	
Within 9 months	19 (36%)	
Time from second and third vaccination, median (IQR), days	230 (214, 241)	255 (252, 259)
Time from third vaccination to serological assessment, median (IQR) days	45 (35, 63)	27 (22, 41)

*DLBCL* *NOS* indicates diffuse large B-cell lymphoma, not otherwise specified, *FL* follicular lymphoma, *IQR* interquartile range, *MM-M* Primary vaccine series with mRNA-1273 followed by booster dose with mRNA-1273, *MM-P* Primary vaccine series with mRNA-1273 followed by booster dose with BNT162b2, *NA* not applicable, *PP-M* primary vaccine series with BNT162b2 followed by booster dose with mRNA-1273, *PP-P* primary vaccine series with BNT162b2 followed by booster dose with BNT162b2, and *WBC* white blood cells.

^a^Other B-cell malignancies included three intravascular large B-cell lymphomas and mantle cell lymphomas, two extranodal marginal zone lymphomas of mucosa-associated lymphoid tissue, and Burkitt lymphoma, primary central nervous system lymphoma, splenic marginal zone lymphoma, and lymphoplasmacytic lymphoma.

^b^Benign hematologic diseases included idiopathic thrombocytopenic purpura and thrombotic thrombocytopenic purpura.

^c^Active therapy included six obinutuzumab maintenance therapies for follicular lymphoma. One patient received acalabrutinib or placebo as maintenance therapy in a clinical trial after rituximab-bendamustine therapy for mantle cell lymphoma.

Humoral responses were evident in 8 (15%) and 22 (42%) patients before and after the third vaccination, respectively. All healthy volunteers remained seropositive before and after their third doses (Fig. 1A, B). T-cell response before booster vaccination was observed in 16 (30%) patients, including 14 seronegative patients (Fig. 1C). After the third vaccination, it was detected in 27 patients (51 %), including 16 seronegative patients (Fig. 1C). In total, while either humoral or cellular responses were identified in 22 (42%) patients after two doses of the vaccine, a third vaccination induced them in 36 (72%) patients (Fig. 1C). In the exploratory subgroup analysis, lower levels of IgM and B cell fractions, previous use of bendamustine, a shorter interval between the last administration of anti-CD20 antibodies, and the third vaccination were associated with poor humoral responses (Table S1). Conversely, the presence of humoral response before the third vaccination was associated with higher titers of anti-S1 antibodies, comparable with those of healthy controls (Fig. 1B, Table S1). In addition, a shorter interval between the last administration of anti-CD20 antibodies and the third vaccination was not associated with the acquisition of T-cell responses (Table S2). Details of adverse events (Fig. S1) are available in the supplementary material.

**Fig. 1:**
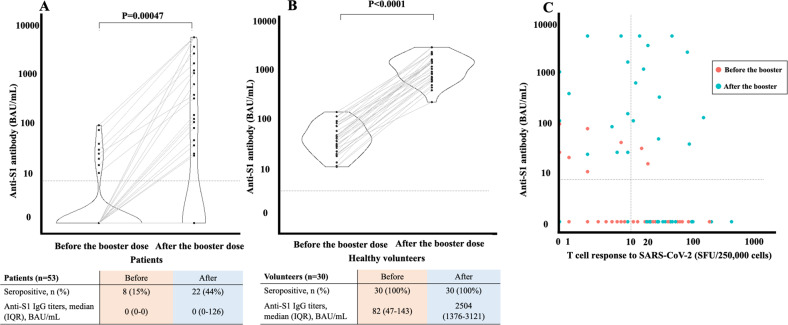
Anti-S1 antibody titers before and after booster vaccination. Booster vaccination in **A** patients and **B** healthy volunteers. *P* indicates probability value. *P* value was calculated via Wilcoxon signed-rank test. **C** Dot plot showing the humoral and cellular responses before and after booster vaccination in patients. Orange and blue dots indicate patients before and after the booster vaccination, respectively. Horizontal dot lines indicate 7.1 BAU/mL, and vertical dot lines indicate 10 SFUs per 250,000 cells and cutoff values of serological and cellular responses, respectively.

Humoral responses are critical for blocking the acquisition of viral infection [9]. In this study, the seropositivity rate before the third vaccination was only 15%, and it improved to 42% after the third vaccination, which is still unsatisfactory but encouraging. In some previous studies, the third vaccination was performed within six months after two doses, and their seroconversion rate tended to be lower, probably due to incomplete B-cell recovery [5, 6]. In our study, we expected that a longer interval (>7 months) would positively affect humoral response by allowing B-cell recovery, resulting in a slightly better seroconversion rate.

In addition, it should be noted that the anti-S1 antibody titers in patients were substantially lower than those in the healthy volunteers. Anti-S1 IgG titers have been reported to be associated with neutralization activity against SARS-CoV-2, and an observational study has indicated that neutralization activity is absent if anti-S1 antibody levels are low [10]. Among patients with a detectable but weak humoral response after administering two doses of an mRNA COVID-19 vaccine, an increase in antibody levels after administering the third dose has been observed [11]. Our study confirmed this finding; patients with a detectable humoral response before the third vaccination exhibited an increase in the anti-S1 antibody titers, comparable with those in healthy controls.

Cellular immune responses most likely control viral replication after infection and prevent progression to severe diseases [9]. The presence of cellular response against SARS-CoV-2 in seronegative patients who recently received anti-CD20 antibodies has been reported [12, 13], indicating some benefits of vaccination even though they failed to exhibit a humoral response. Our study demonstrated that 30% of the patients had T-cell responses against SARS-CoV-2 after two doses of the vaccine. It increased to 51%, three-fifths of whom were seronegative, after the third vaccination. In our study, the interval between the last administration of anti-CD20 antibodies and the third vaccination was not correlated. The prompt, up-to-date administration of mRNA-based COVID-19 vaccines might be beneficial for cellular immunity, even though they failed to achieve seroconversion.

This study has several limitations. First, the sample size was too small to establish the predictors of impaired humoral and cellular responses. Second, the regimens used in combination with the anti-CD20 antibodies were heterogeneous. Therefore, the potential effect of medications other than anti-CD20 antibodies on humoral and cellular responses should be considered. Third, all participants received monovalent COVID-19 vaccines for their third dose. Impaired neutralizing responses induced by the third monovalent COVID-19 vaccines have been reported against the current variants in circulation, such as BQ.1 and XBB [14].

In summary, we confirmed that the third vaccination with an mRNA-based COVID-19 vaccine induces humoral and cellular immune responses in patients who recently received anti-CD20 antibodies. In addition, some seronegative patients achieved seroconversion after the third vaccination. A T-cell response against SARS-CoV-2 was identified after the third vaccination in some patients who failed to gain humoral response, indicating the benefit of prompt, up-to-date vaccination among the vulnerable population. We need more studies to determine the factors associated with impaired vaccine effectiveness and the optimal timing of vaccination in B cell-depleted patients.

## Supplementary information

Supplementary Data

## Data Availability

The datasets generated and/or analyzed during the current study are available from the corresponding author upon reasonable request.
