# Supplementary material for: Humoral and cellular responses after COVID-19 booster vaccination in patients recently treated with anti-CD20 antibodies
Source: Blood Cancer J. 2023 Jan 23;13(1):17. doi: 10.1038/s41408-023-00792-z (PMC9868506; doi:10.1038/s41408-023-00792-z)
Supplement: Supplementary file 1 — Supplementary Data [file 41408_2023_792_MOESM1_ESM.docx]

**Supplementary materials**

**Humoral and cellular responses after COVID-19 booster vaccination in patients recently treated with anti-CD20 antibodies**

Masashi Nishikubo, Yoshimitsu Shimomura, Ryusuke Yamamoto, Satoshi Yoshioka, Hayato Maruoka, Seiko Nasu, Tomomi Nishioka, Kenji Sakizono, Satoshi Mitsuyuki, Tomoyo Kubo, Naoki Okada, Daishi Nakagawa, Kimimori Kamijo, Hiroharu Imoto, Yuya Nagai, Nobuhiro Hiramoto, Noboru Yonetani, Tadakazu Kondo, Chisato Miyakoshi, Asako Doi, and Takayuki Ishikawa

**1. Supplementary methods**

**2. Supplementary tables**

**3. Supplementary figures**

**4. Supplementary references**

**Supplementary methods**

*The kit used for antibody detection*

We used the Abbott Architect SARS-CoV-2 IgG Quant II chemiluminescent microparticle immunoassay kit (Abbott, Sligo, Ireland) to detect IgG antibodies against the receptor-binding domain of the S1 subunit of the spike protein of SARS-CoV-2 (anti-S1 IgG antibody). Anti-S1 IgG antibody concentrations are expressed as binding antibody units per milliliter (BAU/mL). The analytical measurement range of the assay was 7.1–5 680 BAU/mL. To classify participants as seropositive against SARS-CoV-2 spike protein, a threshold of 7.1 AU/mL anti-S1 IgG antibody concentration was set according to the manufacturer’s instructions. We performed the analysis and set the titers of seronegative patients to 0 AU/mL.

*The kit used for SARS-CoV-2-specific T cells*

SARS-CoV-2-specific T cells were detected using an interferon-gamma enzyme-linked immunospot (ELISpot) assay kit (T-SPOT Discovery SARS-CoV-2, Oxfordshire, UK), which has three panels for SARS-CoV-2-specific antigens: the spike, nucleocapsid, and membrane proteins. A positive cellular response to the mRNA vaccine was assessed based on a previous study and the manufacturer’s instructions: number of spots is ≥10 spot-forming units (SFUs) per 250,000 cells in the spike protein panel [1]. We treated patients with a positive reaction to nucleocapsid or membrane protein, defined as ≥10 SFUs per 250 000 cells, as patients with undocumented previous COVID-19 infection and excluded them from this study.

*The investigation other than humoral and cellular responses*

In addition to the anti-S1 IgG antibody, we examined serum immunoglobulin (Ig) levels and conducted analysis of lymphocyte subsets, including CD4-positive and CD8-positive T cells, B cells, and NK cells, in the blood samples collected before booster vaccination.

*Statistical procedures*

Continuous variables were summarized using medians and interquartile ranges (quartiles 1–3), and categorical variables were summarized as counts and percentages. We used the Wilcoxon signed-rank test to compare anti-S1 antibody titers and T cell responses against SARS-CoV-2 before and after booster vaccination. We compared anti-S1 antibody titers after booster vaccination using the Mann–Whitney *U* test in patients and healthy volunteers, as well as among the subgroups. We conducted subgroup analyses for IgG (>700 mg/dL and ≤700 mg/dL), IgA(>800 mg/dL and ≤80 mg/dL), IgM(>40 mg/dL and ≤40 mg/dL), total lymphocytes count (>1.0 × 10^3^/μL and ≤1.0 × 10^3^/μL), CD4-positive T cell count (>0.4 × 10^3^/μL and ≤0.4 × 10^3^/μL), CD8-positive T cell count (>0.4 × 10^3^/μL and ≤0.4 × 10^3^/μL), B cell fractions (>3% and ≤3%), the previous use of bendamustine (yes and no), and interval between the last administration of anti-CD20 antibodies and booster vaccination (>9 months and ≤9 months) [2, 3]. All statistical analyses were performed using R software package (version 4.1.2; R Development Core Team). Statistical significance was set at P < 0.05. In subgroup analyses, we did not adjust P-values for multiplicity because they were considered exploratory.

**Supplementary tables**

Table S1. Exploratory analysis of factors associated with low anti-S1 IgG titers after a third vaccination

| **Characteristics** | **Median titers, BAU/mL (IQRs)** | **P value** |
| --- | --- | --- |
| **Time interval between the last administration of anti-CD20 antibodies and vaccination** |  | <0.001*  <0.001** |
| >9 months, n = 34 | 64,89 (0.00–954.73) |  |
| ≤9 months, n = 19 | 0.00 (0.00–0.00) |  |
| **IgG** |  | 0.075*  0.115** |
| >700 mg/dL, n = 39 | 0.00 (0–270.00) |  |
| ≤700 mg/dL, n = 14 | 0.00 (0.00–0.00) |  |
| **IgA** |  | 0.055*  0.065** |
| >80 mg/dL, n = 38 | 11.52 (0.00-373.06) |  |
| ≤80 mg/dL, n = 15 | 0.00 (0.00–0.00) |  |
| **IgM** |  | 0.004*  0.010** |
| >40 mg/dL, n = 22 | 59.74 (0.00–1,060.24) |  |
| ≤40 mg/dL, n = 31 | 0.00 (0.00–11.52) |  |
| **Lymphocytes** |  | 0.831*  1.000** |
| >1.0 × 10^3^/μL, n = 36 | 0.00 (0.00–176.51) |  |
| ≤1.0 × 10^3^/μL, n = 17 | 0.00 (0.00–108.97) |  |
| **CD4+ T cells** |  | 0.404*  1.000** |
| >0.4 × 10^3^/μL, n = 21 | 0.00 (0.00–388.16) |  |
| ≤0.4 × 10^3^/μL, n = 32 | 0.00 (0.00–55.97) |  |
| **CD8+ T cells** |  | 0.559*  0.777** |
| >0.4 × 10^3^/μL, n = 33 | 0.00 (0.00-82.71) |  |
| ≤0.4 × 10^3^/μL, n = 20 | 0.00 (0.00-273.45) |  |
| **B cell fraction** |  | <0.001*  <0.001** |
| >3%, n = 22 | 357.96 (89.28–2,418.83) |  |
| ≤3%, n = 31 | 0.00 (0.00–0.00) |  |
| **Previous use of bendamustine** |  | 0.031*  0.013** |
| Yes, n = 23 | 0.00 (0.00-0.00) |  |
| No, n = 30 | 31.03 (0.00-283.78) |  |
| **Seropositivity before booster vaccination** |  | <0.001*  <0.001** |
| Yes, n = 8 | 2 176.37 (997.71–5,680.00) |  |
| No, n = 45 | 0.00 (0.00–25.29) |  |
| **Type of vaccines used for the primary two doses** |  | 0.029*  0.028** |
| BNT162b2, n=50 | 0.00 (0.00-121.96) |  |
| mRNA-1273, n=4 | 1 349.03 (33.90-3,415.97) |  |

*P-values were calculated using Mann–Whitney *U* test.

** P-values were calculated by Chi-square test.

Table S2. Exploratory analysis of factors associated with impaired T cell responses against SARS-CoV-2 after a third vaccination

| **Characteristics** | **Proportion of human participants who acquired positive T cell responses against SARS-CoV-2, n (%)** | **P value** |
| --- | --- | --- |
| **Time interval between the last administration of anti-CD20 antibodies and vaccination** |  | 1.000 |
| >9 months, n = 34 | 17 (50.0%) |  |
| ≤9 months, n = 19 | 10 (52.6%) |  |
| **IgG** |  | 0.224 |
| >700 mg/dL, n = 39 | 22 (56.4%) |  |
| ≤700 mg/dL, n = 14 | 5 (35.7%) |  |
| **IgA** |  | 0.766 |
| >80 mg/dL, n = 38 | 20 (52.6%) |  |
| ≤80 mg/dL, n = 15 | 7 (46.7%) |  |
| **IgM** |  | 0.271 |
| >40 mg/dL, n = 22 | 9 (40.9%) |  |
| ≤40 mg/dL, n = 31 | 18 (58.1%) |  |
| **Lymphocytes** |  | 1.000 |
| >1.0 × 10^3^/μL, n = 36 | 18 (50.0%) |  |
| ≤1.0 × 10^3^/μL, n = 17 | 9 (52.9%) |  |
| **CD4+ T cells** |  | 0.264 |
| >0.4 × 10^3^/μL, n = 21 | 13 (61.9%) |  |
| ≤0.4 × 10^3^/μL, n = 32 | 14 (43.8%) |  |
| **CD8+ T cells** |  | 0.158 |
| >0.4 × 10^3^/μL, n = 33 | 14 (42.4%) |  |
| ≤0.4 × 10^3^/μL, n = 20 | 13 (65.0%) |  |
| **B cell fraction** |  | 1.000 |
| >3%, n = 22 | 1 (50.0%) |  |
| ≤3%, n = 31 | 14 (51.6%) |  |
| **Previous use of bendamustine** |  | 0.785 |
| Yes, n = 23 | 11 (47.8%) |  |
| No, n = 30 | 16 (53.3%) |  |
| **Seroconversion before booster vaccination** |  | 1.000 |
| Yes, n = 8 | 4 (50.0%) |  |
| No, n = 45 | 23 (51.1%) |  |

P-values were calculated via Chi-square test.

**Supplementary figures**


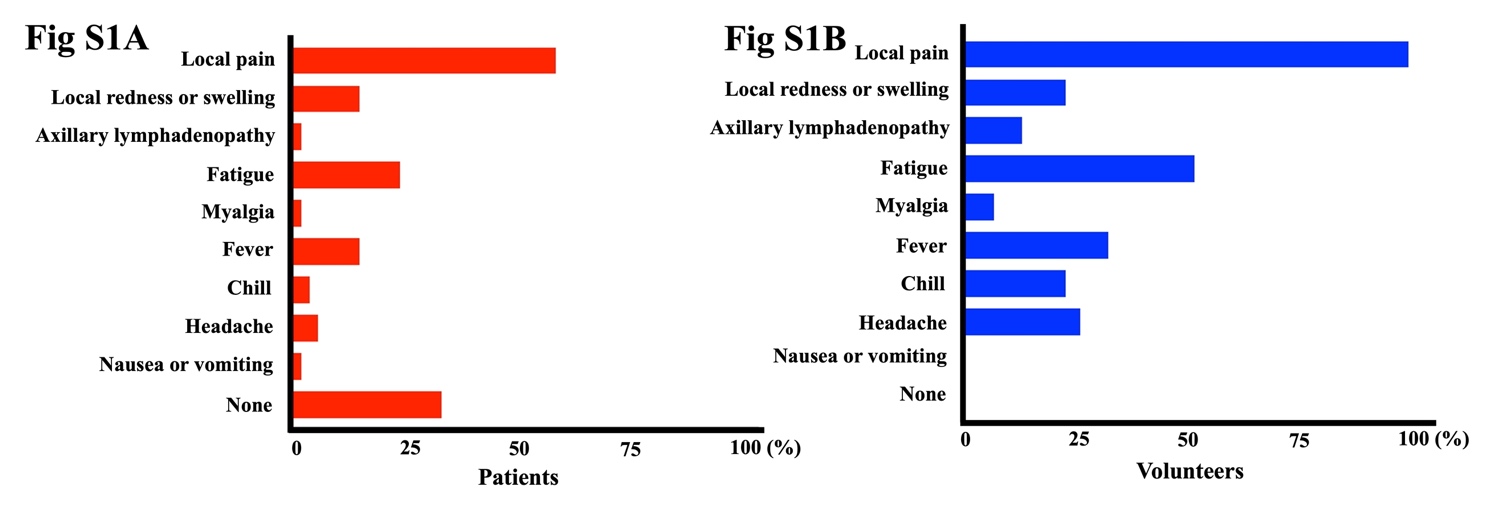


**Figure S1.** **Adverse events reported by (A) patients and (B) healthy controls**. All evaluable patients and healthy volunteers reported the adverse events after the third vaccination in a questionnaire. Adverse events were defined as local pain, local redness or edema, axillary lymphadenopathy, fatigue, myalgia, fever, chills, headache, nausea or vomiting, and anaphylaxis. The patients and healthy volunteers commonly reported local pain at the injection site, fatigue, and fever. After the third vaccination, local pain at the injection site, fatigue, and fever were more commonly reported in the healthy volunteers (100%, 52%, and 32%, respectively) than in the patient group (59%, 24%, and 15%, respectively). All adverse events were mild and spontaneously resolved. Anaphylaxis was not reported.

**Supplementary references**

1. Prendecki M, Thomson T, Clarke CL, Martin P, Gleeson S, De Aguiar RC, et al. Immunological responses to SARS-CoV-2 vaccines in kidney transplant recipients. Lancet. 2021; 398:1482–1484.

2. Lim SH, Stuart B, Joseph-Pietras D, Johnson M, Campbell N, Kelly A, et al. Immune responses against SARS-CoV-2 variants after two and three doses of vaccine in B-cell malignancies: UK PROSECO study. Nat Cancer. 2022; 3:552–564.

3. Nishikubo M, Shimomura Y, Maruoka H, Nasu S, Nishioka T, Sakizono K, et al. Humoral response and safety of the BNT162b2 and mRNA-1273 COVID-19 vaccines in patients with haematological diseases treated with anti-CD20 antibodies: an observational study. Br J Haematol. 2022; 197:709–713.
